# The Minamata Convention on Mercury: A First Step toward Protecting Future Generations

**DOI:** 10.1289/ehp.121-A304

**Published:** 2013-10-01

**Authors:** Rebecca Kessler

**Affiliations:** Rebecca Kessler is a science and environmental journalist based in Providence, RI.

In July 1956, in a fishing village near the city of Minamata on Japan’s Shiranui Sea, a baby girl named Shinobu Sakamoto was born. Her parents soon realized something was wrong. At 3 months old, when healthy babies can hold up their heads, Sakamoto could not. She grew slowly and began crawling unusually late. At age 3 years, she drooled excessively and still couldn’t walk. Her parents sent her to live at a local hospital, where she spent four years in therapy to learn to walk, use her hands, and perform other basic functions. Early on, several physicians agreed on a diagnosis of cerebral palsy.

Yet there were signs that Sakamoto’s condition was part of something much bigger. A few years before her birth, dead fish and other sea creatures had begun appearing in Minamata Bay.^1^ Seabirds were losing their ability to fly.^2^ And cats were dying off, many from convulsions that locals called “dancing disease.”^1^ Then, two months before Sakamoto’s birth, an outbreak of an unknown neurological illness was first reported among the area’s fishing families. Sakamoto’s older sister, Mayumi, and several of the family’s neighbors were diagnosed with the mysterious ailment, which was attributed to contaminated seafood. In 1957 scientists gave the ailment a name: Minamata disease. The next year, Mayumi died of it.

**Figure 1:**
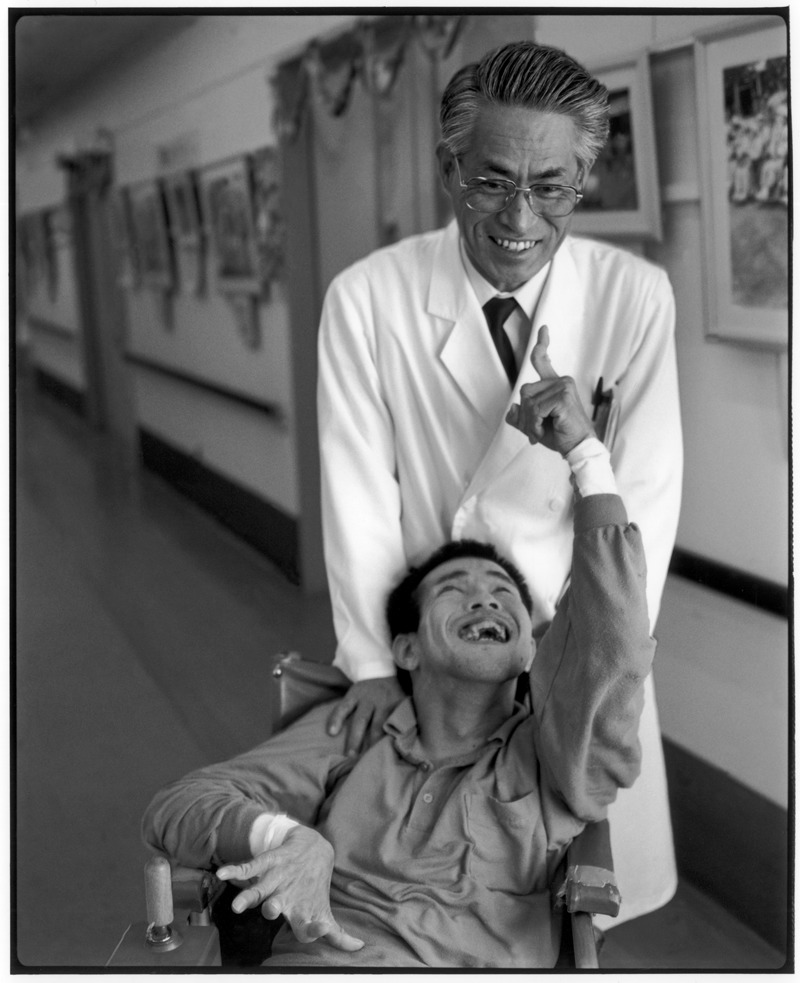
Dr. Hiroyuki Moriyama and Kazumitsu Hannaga, a congenital Minamata disease patient, at Meisui-en Hospital, Minamata, 1991. The hospital opened in 1972 to care for Minamata victims.^31^ © Chris Steele-Perkins/Magnum Photos

The responsible contaminant was eventually identified as methylmercury that had been discharged in wastewater from a local chemical factory owned by the Chisso Corporation.^3^ The numbers of the stricken climbed and spread around the Shiranui Sea, and in 1962 a cluster of children originally diagnosed with cerebral palsy, including Sakamoto, were recognized as suffering from congenital Minamata disease. But the government did nothing to stop Chisso’s dumping or to discourage people from eating fish, and only acknowledged the plant’s role in Minamata disease after it stopped using mercury on its own. That was 1968. By then Sakamoto was 12.

The Minamata disaster—which affected thousands of individuals, including every member of Sakamoto’s family—was the first large-scale incident of methylmercury poisoning. But it wasn’t the only one. A similar, smaller methylmercury poisoning incident in Niigata Prefecture came to light in 1965, as did another among Native Americans in Ontario, Canada, in 1969.^4^^,^^5^

Decades after industrial dumping ceased, thousands of survivors of these incidents are still suffering from a host of neurological symptoms, including tremors, dizziness, headaches, memory loss, and vision and hearing problems; the most severe cases also involve developmental disabilities, cognitive and motor dysfunction, and physical abnormalities. “Minamata disease is not over yet,” Sakamoto says today.^6^ At 57, her hands are twisted, and she can no longer walk or bathe without help. She has never been able to work, although she has spent decades advocating on behalf of Minamata victims.

Minamata drew the world’s attention to the devastating effects of mercury, a powerful neurotoxicant now known to be particularly dangerous to fetuses, infants, and young children. Before Minamata, the placenta was thought to protect the fetus against toxicants.^2^

But even less severe mercury pollution is now known to be problematic. “We started with Minamata fifty years ago, and now we know that doses that we thought were safe in the past are certainly not safe,” says Philippe Grandjean, an environmental health scientist at the Harvard School of Public Health and the University of Southern Denmark. “We’re now concerned about exposures that are highly prevalent in seafood consumers worldwide.”

In October 2013 a new international convention to control mercury emissions will be open for signing in Japan. Named the Minamata Convention on Mercury, the agreement is a response to the realization that mercury pollution is a global problem that no one country can solve alone. The convention was four years in the making, with more than 130 nations agreeing by consensus to a final text in January 2013. It includes both compulsory and voluntary measures to control mercury emissions from various sources, to phase the element out of certain products and industrial processes, to restrict its trade, and to eliminate mining of it.^7^

## Sources of Mercury

Mercury is a naturally occurring element used in numerous products and industrial processes, from thermometers and certain bulbs to chemical catalysts. It is released by the burning of fossil fuels and the production of cement and some metals.^8^

Human activities are estimated to have released 1,960 metric tons of mercury into the atmosphere and at least 1,000 metric tons into the water in 2010, according to a 2013 report by the United Nations Environment Programme (UNEP).^8^ The report notes that after a period of apparent stability between 1990 and 2005, global emissions to the air may be rising again in some sectors.

Rapidly industrializing Asia is the largest current source of atmospheric mercury emissions, with China contributing a third of the global total.^8^ Meanwhile, countries in Europe and North America have cut air emissions substantially. The United States has done so in part by cleaning up waste incinerators. And power plants have until 2016 to comply with new federal standards that will dramatically limit mercury and other pollutants in their emissions.^9^ But mercury tends to linger in the environment, and a recent modeling study estimated that half the mercury pollution in the surface layer of the ocean today came from emissions prior to 1950, when U.S. and European contributions exceeded those from Asia.^10^

That study also projected that if mercury emissions stopped altogether in 2015, atmospheric deposition levels would drop immediately by 30%—good news. After that, however, the decline would slow, and it would take an estimated 85 years—until 2100—for atmospheric deposition to drop by about half and for ocean-surface levels to drop by one-third. And that forecast does not account for climate change, which may complicate things, for instance by thawing northern tundra and releasing long-stored mercury back into circulation. The authors concluded that even aggressive emissions cuts will merely maintain current mercury levels in the ocean.^10^

Humankind is now known to have released much more mercury into the environment than previously thought. Atmospheric levels are now more than seven times higher and ocean-surface levels are nearly six times higher than they were around 2000 b.c., which is roughly when human-caused emissions are believed to have begun.^10^

Mercury emissions can travel far from their original sources on winds and ocean currents.^11^ Once mercury lands in soils and waterways, microorganisms metabolize it into methylmercury, the element’s most toxic form, which accumulates up the food chain.^12^ People are typically exposed to methylmercury when they eat contaminated seafood. Mercury concentrations in human hair collected from numerous contamination hot spots identified around the world indicate people in these areas regularly eat fish considered unsafe by U.S. Environmental Protection Agency standards, according to a 2013 report by the Biodiversity Research Institute and the International POPs Elimination Network (IPEN).^13^ David Evers, chief scientist at the Biodiversity Research Institute, says that study, although small, is unique in its geographic breadth. However, the critical question of how prevalent mercury exposure is in people around the globe remains unanswered.

**Figure 2:**
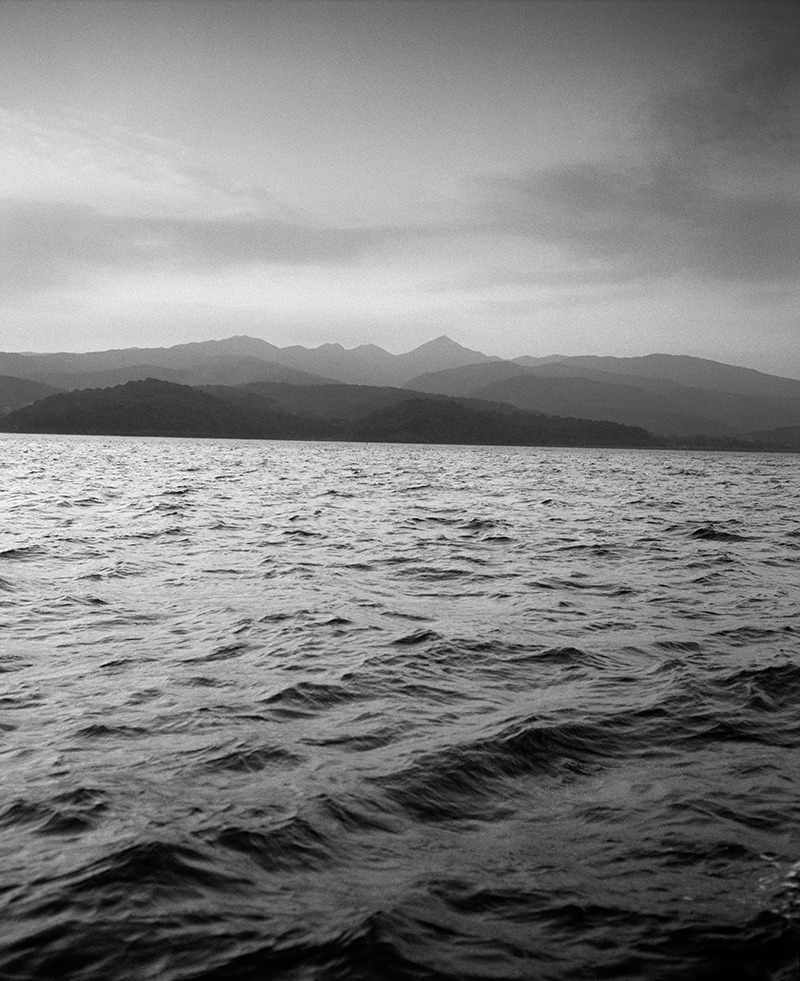
Minamata Bay in 1991. In the 12 years between the time that Minamata disease was first identified and Chisso Corporation stopped producing acetaldehyde, the company dumped another 80–150 tons of methylmercury into the harbor.^3^ © Chris Steele-Perkins/Magnum Photos

Extensive research documents methylmercury’s developmental toxicity.^14^ Women showing few symptoms of methylmercury exposure can still pass devastating doses along to their unborn children, as the Minamata case shows.^15^ In Minamata, residents’ median hair-mercury level was 30 ppm.^2^ But several studies suggest that children exposed even to low doses *in utero* may be at risk for various neuropsychological problems.^14^ For instance, in one study, children born to mothers with hair-mercury levels of just 1 ppm had an increased risk of behaviors related to attention deficit/hyperactivity disorder when they were 8 years old.^16^

One recent study by Grandjean and colleagues calculated that within the European Union, at least 1.8 million children with elevated methylmercury exposure are born each year, resulting in an annual loss of more than 600,000 IQ points and up to ¤9 billion ($11.9 billion) in associated economic productivity.^17^ Methylmercury also affects the health of fish stocks themselves, threatening an essential food supply for millions of people and other piscivorous animals.^18^

## Toward an Agreement

Global support for a binding agreement on mercury emissions began building in 2003. But the United States pushed instead for voluntary measures to control emissions, making a legally binding convention a nonstarter.^19^ Then, at a 2009 UNEP Governing Council meeting shortly after Barack Obama’s inauguration, the United States announced it would move ahead with negotiations toward a binding instrument.

The Governing Council quickly established the negotiating process. Its backbone was a series of five meetings where delegates from participating nations hashed out the convention text, with substantial input and lobbying from nonvoting outside groups.

Daniel Reifsnyder, deputy assistant secretary for environment with the State Department, oversaw the U.S. role in the negotiations. Pointing to the Stockholm Convention on Persistent Organic Pollutants and other international agreements restricting hazardous chemicals that the United States has not joined, he says, “We were concerned in this case not to go down a well-worn path that leads us to negotiate but then not be able to implement, so negotiating something meaningful yet flexible was key.”

At the first negotiating session, held in Stockholm in 2010, representatives from IPEN and a Swedish nongovernmental organization tested the hair of participants from 40 countries for mercury. Every sample came back positive, and more than a third exceeded the U.S. National Research Council reference dose of 1,000 µg/kg (1 ppm). Mercury levels of participants from poorer countries averaged 1,182 µg/kg, and those from wealthier countries averaged 669 µg/kg. One sample topped the charts at more than 20,000 µg/kg.^20^

These results effectively lifted the veil of abstraction that often shrouds diplomatic talks, says Joseph DiGangi, a senior science and policy advisor with IPEN. “When delegates actually found out that the topic of the negotiation was in their own body, quite a few of them came up and just couldn’t believe it,” he says. “They said, ‘What is it doing in me?’”

Fast-forward two and a half years and three more negotiating sessions. Exhausted delegates emerged from all-night discussions at the fifth and final negotiations in Geneva and officially adopted the convention at 7:00 a.m. on 19 January 2013. By all accounts, one of the most important and difficult topics to settle was how to control air emissions of mercury from facilities such as waste incinerators, smelters, and coal-fired power plants. The power plants were central to the discussion because they are the world’s second biggest mercury source, releasing 24% of global emissions.^8^ Yet large developing nations depend on cheap national coal supplies to bring electricity to their citizens and fuel their growing economies.

Some developing nations initially balked at the cost of technology that removes mercury from smokestack emissions. The United States worked hard to convince China and India, in particular, that mandatory controls in this sector could be affordably achieved through the application of so-called best available techniques, according to the State Department’s Reifsnyder. In the end, the United States succeeded, although a detailed description of acceptable techniques remains to be worked out, and they are required only for new sources of mercury air emissions. Reifsnyder describes the result as “robust enough to be meaningful, yet flexible enough to be implementable.”

Aleksandra Tomczak, policy manager for the World Coal Association, who attended three negotiations, also came away satisfied. “It actually does, in our opinion, strike a balance between environmental protection priorities and development objectives,” she says.

However, critics like IPEN’s DiGangi say that although the measure should reduce the mercury emissions per unit of energy produced, countries are free to keep building capacity, so total emissions will probably rise. “The treaty will address some mercury sources—it just will not be able to keep up with the increased mercury emissions,” DiGangi says. “In other words, it will change the slope, but the amount of mercury pollution will still increase.”

Critics also say the time frame for implementation is too long. Countries have 5 years before they must build new sources that comply and 10 years before they must at least establish a goal to reduce emissions from existing sources. But the clock doesn’t start ticking until the convention is ratified, which is unlikely for another few years, says Susan Egan Keane, a senior environmental analyst with the Natural Resources Defense Council. “You’re basically grandfathering in … thousands of tons of mercury emitted during that time that you’re sitting around not doing anything,” Keane says. “That’s a lot of mercury that you’re just letting go!”

**Figure 3:**
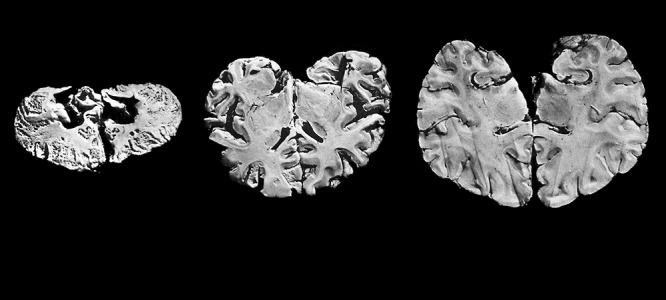
Samples of brain tissue from Minamata disease patients (left and center) illustrate the devastating effect of high methylmercury exposure. The sample on the left is from a 7-year-old child who died after four years of exposure, the sample in the middle is from an 8-year-old child who died after 2.75 years of exposure, and the sample on the right is from a healthy 30-year-old man. © Robin Treadwell/Science Source

Another key issue was the biggest source of mercury pollution, artisanal and small-scale gold mining, which accounts for more than a third of global emissions. Small, often temporary mining operations have boomed worldwide as the price of gold skyrocketed. Some 10–15 million people, including possibly as many as 3 million women and children, many of them extremely poor, are estimated to work in the industry.^21^

To separate tiny particles of gold from ore, workers commonly use large quantities of mercury with no protection whatsoever for themselves, their homes, or the environment. According to Keane, mercury is cheap and readily available to miners. She recalls visiting a mine in Borneo where she watched a worker casually amalgamate gold with mercury he poured from a soda bottle. She later calculated the bottle held roughly as much mercury as 60,000 compact fluorescent light bulbs; she says the miner may well have used a bottle each day. [For more information, see “Quicksilver and Gold: Mercury Pollution from Artisanal and Small-Scale Gold Mining” in the November 2012 issue of *EHP*.^22^]

Various countries have tried outlawing mercury in artisanal and small-scale gold mining, but without help for miners to transition away from the practice, it has simply gone covert, Keane says. She says the convention took the right approach by directing countries to come up with their own plans to reduce or eliminate mercury in mining. Guidelines for the plans mandate strategies to formalize the industry and eliminate its most polluting practices, and to protect children and pregnant women from mercury exposure. However, the convention allows continued mercury trade for artisanal and small-scale gold mining, and there is no phase-out date for the practice.

The convention does phase out mercury in most products by 2020, including pesticides and certain batteries, bulbs, switches, cosmetics, and measuring devices. One product that sparked extensive debate was dental amalgam. The Zero Mercury Working Group, a coalition of environmental and health organizations, led the charge to include amalgam in the convention by pointing out that it is a significant source of mercury emissions from cremated human remains and amalgam waste washed down the drain.^23^ The convention “phases down”—gradually reduces but doesn’t eliminate—the use of mercury-containing dental amalgam by directing countries to adopt at least two control measures from a list of nine options.

Another hotly debated product was the mercury-based vaccine preservative thimerosal. Although thimerosal has been eliminated from most children’s vaccines in developed nations, it is still widely used throughout the developing world because it enables vaccines to be packaged in multidose bottles, significantly lowering costs and making it easier to transport and distribute vaccines in remote areas.

Two U.S. organizations, SafeMinds and the Coalition for Mercury-Free Drugs, pushed for the convention to phase out or phase down thimerosal, contending that it poses a risk to children’s health.^24^^,^^25^ Numerous global health agencies led by the World Health Organization rallied to protect it, however, arguing that the preservative is safe and essential to vaccination programs that protect the world’s poorest children from life-threatening diseases.^26^ A number of developing nations expressed concern about thimerosal during the negotiations, but in the end they supported its continued use, and the convention specifically exempts it.

The convention also addressed specific manufacturing processes, notably phasing out mercury in the production of acetaldehyde, the source of the contamination at Minamata. By 2020 countries must halve the use of mercury in the production of vinyl chloride monomer, the main component of PVC plastic. China is all but alone in manufacturing vinyl chloride monomer in a way that uses mercury as a catalyst, but IPEN describes the Chinese industry as an unquantified and “potentially enormous” emissions source.^27^

**Figure 4:**
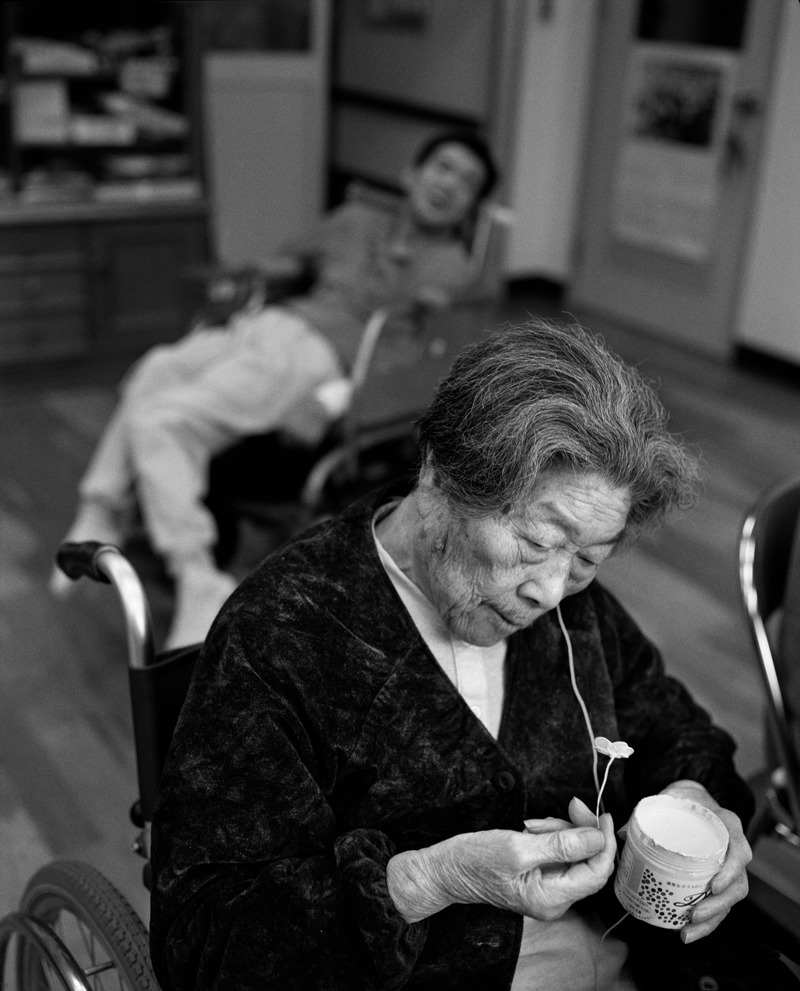
In Meisui-en Hospital, an elderly Minamata disease patient makes plastic flowers as occupational therapy, 1991. Decades after dumping ceased, victims continue struggle with debilitating symptoms of mercury poisoning. © Chris Steele-Perkins/Magnum Photos

Developing nations drove the negotiations on two other points of contention. One was the inclusion of an article devoted to health issues. Developed nations opposed including one, largely out of concern that it would open the door for costly public health programs to be included in the convention, according to Keane. The final convention does include a health article, albeit a brief one simply encouraging nations to implement general measures to protect their populations from mercury exposure.

Developing nations were also concerned about securing enough international funding to implement the convention effectively. After much discussion, the final convention designates the Global Environment Facility Trust Fund as the funding mechanism, but it remains to be determined how much donor countries will give to the fund or, therefore, how much recipient countries will receive. “The treaty is one thing, but now implementing it is also another process, which will bring on board a lot of other issues—capacity, capability, resources, and understanding,” says Richard Mwendandu, a delegate from Kenya.

Yet the convention has drawn praise, even from some critics, as an important first step and the first unified global action to curb mercury emissions. “The treaty involved compromises, but it reflects a global consensus that mercury emissions and releases represent a serious health and environmental concern,” says Evers, of the Biodiversity Research Institute.

## Bringing the Convention Home to Minamata

The Japanese government pushed for the convention to be named after the Minamata tragedy.^28^ Even so, nearly 60 years after that incident came to light, victims’ groups say the Chisso Corporation has not been held sufficiently accountable, and the pollution has not been properly cleaned up. And they say the Japanese government has neither fully assessed the damage to human health and the environment nor adequately compensated victims.

The government officially recognizes fewer than 3,000 patients from the Minamata and Niigata incidents, more than half of whom are now dead. Those patients have received some compensation and medical expense payments, while around 10,000 others have received more modest compensation for having “applicable conditions.”^29^ Yet more than 65,000 people have reportedly applied for compensation and medical expenses under a new program.^30^

**Figure 5:**
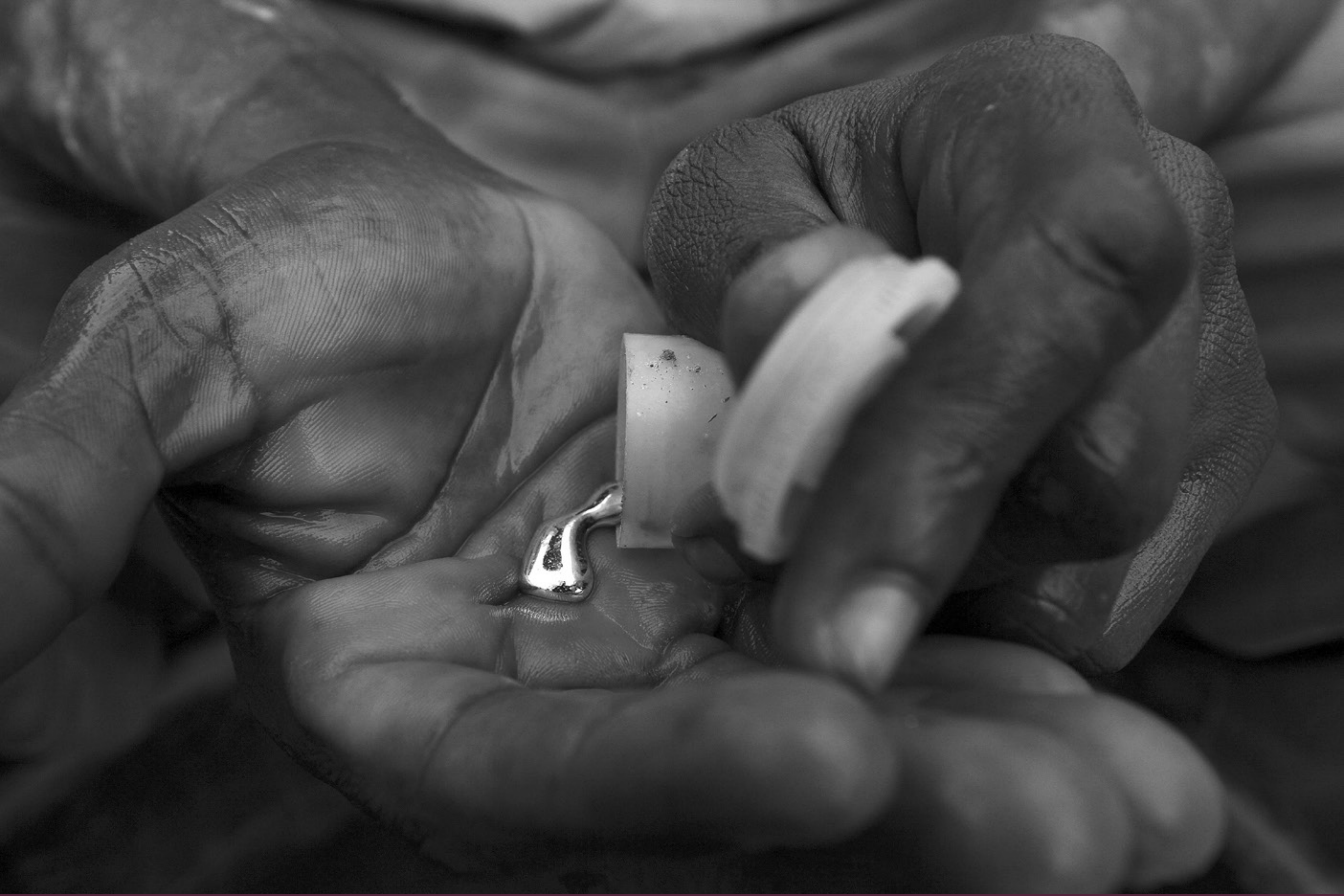
A miner in Obuasi, Ghana, holds liquid mercury in his hand, 2009. Artisanal and small-scale gold mining currently accounts for more than a third of global mercury emissions. © George Osodi/Panos Pictures

During the negotiations, several Minamata disease victims’ groups and other organizations argued that if the convention was to bear the Minamata name, the Japanese government must resolve these issues at home, and the convention should be strong enough to prevent similar tragedies. Shinobu Sakamoto traveled to Chiba, Japan, for the second negotiating meeting in January 2011 to make brief statements supporting that message. The final convention left the groups disappointed. Takeshi Yasuma, coordinator of the Tokyo-based Citizens Against Chemicals Pollution, lobbied extensively on the issue. “The naming profanes the honor of the victims in Minamata,” he says.

Before the agreement can enter into force, at least 50 nations must ratify it, a number Reifsnyder says isn’t expected to be reached until approximately 2017. Meanwhile, however, nations will sign the convention starting this month in Japan and will begin to enact any legislation necessary to comply with it. At press time, Reifsnyder says the U.S. State Department is conducting “the customary process of evaluating the convention to assess how the United States would implement its obligations if it were to join”—a precursor to a formal decision on whether the United States will join.

Once the convention enters into force, only a strong program for monitoring mercury in the environment, in wildlife, and especially in people will tell how well it is accomplishing its fundamental purpose of reducing human exposure, says Evers. The convention outlines the bones of such a monitoring program, which Evers and other scientists are working to flesh out. However, he says the human monitoring component remains uncertain, with some countries expressing concern about costs, logistics, and their potential liability for caring for people found to have dangerous levels of mercury.

While hailing the convention as a landmark achievement, Harvard’s Grandjean says he hopes countries will go beyond its mandates, particularly when it comes to protecting children’s health with measures such as provision of dietary advice and routine screening of pregnant women for mercury exposure.

But even if the convention successfully reduces new emissions, we will be stuck with the mercury already in the environment for a long time. “Mercury concentrations in tuna or in swordfish are not going to decrease in the short term,” Grandjean says. “This is going to take perhaps even centuries.”
